# Improving the Quality of Medical Documentation in Orthopedic Surgical Notes Using the Surgical Tool for Auditing Records (STAR) Score

**DOI:** 10.7759/cureus.19193

**Published:** 2021-11-01

**Authors:** Baraa Mafrachi, Abdallah Al-Ani, Ashraf Al Debei, Mohamad Elfawair, Hussien Al-Somadi, Mohammed Shahin, Yazan Alda'as, Jihad Ajlouni, Amjad Bani Hani, Mahmoud Abu Abeeleh

**Affiliations:** 1 Orthopaedics and Trauma, The University of Jordan, Amman, JOR; 2 Department of Research, King Hussein Medical Center, Amman, JOR; 3 School of Medicine, The University of Jordan, Amman, JOR; 4 Orthopaedics, Jordan University Hospital, Amman, JOR; 5 General Surgery, Jordan University Hospital, Amman, JOR; 6 General Surgery, The University of Jordan, Amman, JOR

**Keywords:** quality, surgical notes, star score, jordan, electronic medical records, electronic health records

## Abstract

Aims

Due to the significant value held by medical records in terms of influencing patient care and medico-legal cases, this study aimed to investigate the quality of surgical notes and their improvement through periodic auditing during a six-year period at a major tertiary hospital.

Methodology

This study retrospectively evaluated surgical records of patients undergoing elective orthopedic surgeries at Jordan University Hospital from 2016 to 2021 using the Surgical Tool for Auditing Records (STAR) validated questionnaire. This questionnaire is composed of six distinct sections aimed to quantify the quality of medical records and demonstrate their associated deficiencies. Pre- and post-audit STAR scores were analyzed using the two independent sample t-test on Statistical Package for Social Sciences (SPSS) version 23.0 (IBM Corp. Armonk, NY).

Results

A total of 454 records were randomly selected and evaluated using the STAR questionnaire. There was an overall significant trend of improvement in the quality of records in all evaluated years compared to the 2016 baseline. The most pronounced improvements were in the records of 2021 as compared to the 2016 baseline (97.4 ± 0.7 vs. 94.3 ± 1.6; p:<0.05), in which the Initial Clerking, Subsequent Entries, and Operative Record domains had the most significant magnitude of change. The Consent and Anesthesia domains plateaued over the study’s period in terms of overall quality. The most improved STAR domain was the Discharge Summary domain, in which four subsections (follow-up, diagnosis, complications, and medications on discharge) had significant STAR score increases (all; p:<0.05).

Conclusion

Our study implies that simple measures, including personnel education and training and periodic auditing, are effective measures in increasing the quality of surgical records. High-quality medical records need to be sustained and continuously improved, as they contribute to better health care, promote research, and contribute to economic gains through cost-effective practices.

## Introduction

The quality of care provided by any health care institution relies heavily on the integrity, comprehensiveness, and accuracy of the health information recorded within that institution [1]. Health records contain data that is integral to the development of proper patient management, promote clear communication between different health care providers, and form the basis of many types of research [2-4]. It is thus of paramount importance that high standards of medical note recording are maintained amongst the clinical staff [5]. Operative notes, in particular, form a vital component of the postoperative care of patients [6]. Since they are the only legal record of the surgery performed on a patient, they are regarded as obligatory constituents of health care records [7-8].

Paper-based documentation has been viewed as a subpar modality of archiving health care data, presenting issues of incompleteness, illegibility, and redundancy [9]. The incorporation of health information technologies brought upon by electronic medical records (EMR) is proposed to improve healthcare quality and advocate safer medical practice [10-11]. This is brought upon by better adherence to clinical guidelines through more accurate documentation and the design of a clear structure to the medical records [12]. However, recent studies have reported results that conflict with this vision, highlighting a lack of congruence between the integration of EMR and improvement in patient outcomes [13-14]. This discrepancy may be attributed to errors in handling the data, which would compromise the integrity of the information operated by the interface. The dominant prevalence of EMR across various settings foreshadows a compelling impact on the quality of healthcare provided on a wide scale, especially given the concerns arisen [15].

Audits and feedback are recognized as elemental in the strategy that aims to improve the quality, support performance, and safety of health care standards [16-17]. They compare current practices in a certain aspect of a health care system to the guidelines, as such providing bases for education and management in the fields of cost-effectiveness, quality control, and management of risks and resources [18]. Reasons as to why the system fell short are identified as part of the audit, and changes are implemented to rectify these flaws, intending to meet the standards set with the resources available [19]. Regular audits and reviews of performance are viewed as some of the more effective strategies in upholding the quality of medical records, hence serving as valuable means to excellent patient care, and resource allocation [20]. Therefore, this study aims to investigate the quality of surgical records in a major tertiary hospital over a six-year period to demonstrate the impact of periodic audits on the overall comprehensiveness of medical records.

## Materials and methods

We conducted a retrospective evaluation and audited the quality of surgical records using the Surgical Tool for Auditing Records (STAR) tool [21]. The STAR tool is originally designed based on the Royal College of Surgeons’ guidelines on medical record keeping. The tool is composed of 50 components allocated into six domains of different weight allotments, including Initial Clerking (10 items; 20%), Subsequent Entries (8 items; 16%), Consent (7 items; 14%), Anesthetic record (7 items; 14%), Operative record (9 items; 18%), and Discharge summary (9 items; 18%). The total score for each evaluated note is calculated based on the following formula [(50 - deducted points) x 2]. The domain Subsequent Entries is calculated by averaging out the final score over the number of up to four entries post the initial admission. Similarly, the total STAR score is the average of all evaluated notes. Based on the assessment conducted by the score’s developers, the STAR score is a highly reliable evaluation tool (Cronbach α: 0.959), which requires a minimum of 20 records to start an audit [21].

The study included surgical records for patients undergoing orthopedic surgeries at Jordan University Hospital (JUH), Amman, Jordan. JUH is the first and largest academic hospital in Jordan with a total of 600 beds [22]. In addition, it is the major referral center for all of central Jordan serving more than four million patients [23]. JUH treats more than half a million patients annually and conducts about 25,000 surgical operations every single year [24]. According to the Joint Commission International (JCI) accreditation rating, JUH is ranked number one and 18th at the Middle Eastern and global levels in terms of quality excellence, respectively. As of 2009, JUH adopted its own Electronic Health Record (EHR) system, which acted as an adjunct to the hospital’s paper-based system until this current moment.

A random sample of surgical records was chosen among the hospital’s charts from 2016 to 2021. We included records of patients who underwent orthopedic surgery and were admitted for at least one day. Patients undergoing day surgeries or those with records but who had their surgeries delayed were excluded from the study’s sampling. In order to initiate the audit, a pilot assessment was conducted on 20 records to train two authors on how to assess surgical records using the STAR tool and identify areas of possible disagreement in STAR’s concepts interpretation. The two authors independently evaluated 454 surgical records archived within the hospital’s system and then were cross-matched. Any dispute between the two authors was resolved by a final decision from a third senior author blinded to the intent of the evaluation process.

On an annual basis, all medical practitioners among all the 64 specialties in JUH are trained and instructed on how to improve notes through appropriate documentation and clear note-taking. The audit aimed to evaluate the progress of the quality of surgical notes by comparing their total STAR scores and percentages of deficiency within specific areas of note-taking. During the duration of the study, the research team would advocate proper note-taking and better documentation through detailed slides that are based on the recommendations by the Royal College of Surgeons of England. The process is conducted once per year. The collected variables were reported as frequencies [n (%)] and means ± standard deviations wherever applicable. Pre- and post-audit total STAR and section-specific STAR scores were compared using the two independent sample student’s t-test. A p-value of less than .05 at a confidence interval of 95% was considered statistically significant. All data cleaning and statistical analysis were conducted on Statistical Package for Social Sciences (SPSS) version 23.0 (IBM Corp. Armonk, NY).

The study’s protocol was reviewed and accepted by the JUH’s Institutional Review Board (IRB) and the University of Jordan’s research ethics committee. The processes within the study’s protocol conform to the guidelines of the Declaration of Helsinki (as revised in 2008).

## Results

The study included 454 surgical records of patients undergoing orthopedic surgery throughout the following years: 2016 (81), 2017 (82), 2018 (79), 2019 (85), 2020 (85), and 2021 (41). The mean total STAR score for the years 2016, 2017, 2018, 2019, 2020, and 2021 were 94.3 ± 1.6, 95.5 ± 1.7, 95.9 ± 0.8, 95.7 ± 1.1, 96.8 ± 1.2, and 97.4 ± 0.7, respectively. Table 1 demonstrates the overall STAR score and section-specific STAR scores for all the included years.

**Table 1:** STAR scores among surgical records from 2016 to 2021 STAR: Surgical Tool for Auditing Records

	2016	2017	2018	2019	2020	2021
Initial clerking score	95.4 ± 4.7	95.7 ± 3.6	96.3 ± 2.7	96.9 ± 2.8*	98.2 ± 4.3*^!#^	99.7 ± 0.8*^!#$^
Subsequent entries score	88.8 ± 5.7	94.2 ± 1.5*	94.0 ± 1.3*	91.2 ± 5.1*^!^	94.1 ± 4.1*^#^	95.5 ± 2.2*^!#$^
Consent score	95.2 ± 2.8	97.0 ± 2.3*	97.6 ± 0.9*	97.8 ± 0.6*	97.8 ± 0.7*	97.8 ± 0.6*
Anesthetic record score	97.7 ± 1.5	97.5 ± 2.3	97.8 ± 0.8	97.7 ± 1.0	97.9 ± 0.4	97.6 ± 1.9
Operative record score	94.9 ± 1.9	94.6 ± 2.3	94.6 ± 2.6	95.3 ± 2.1	96.3 ± 1.2*^!#^	96.8 ± 1.1*^!#$^
Discharge summary score	93.8 ± 1.9	94.2 ± 2.2	94.9 ± 1.1*	95.3 ± 1.3*^!^	96.5 ± 1.7*^!#^	97.2 ± 1.9*^!#^
Total score	94.3 ± 1.6	95.5 ± 1.7*	95.9 ± 0.8*	95.7 ± 1.1*	96.8 ± 1.2*^!#^	97.4 ± 0.7*^!#$^
* Identifies a significant mean difference when compared to the 2016 baseline at p-value < 0.05						
^!^ Identifies a significant mean difference when compared to the 2018 baseline at p-value < 0.05						
^#^ Identifies a significant mean difference when compared to the 2019 baseline at p-value < 0.05						
^$^ Identifies a significant mean difference when compared to the 2020 baseline at p-value < 0.05						

**Figure 1:**
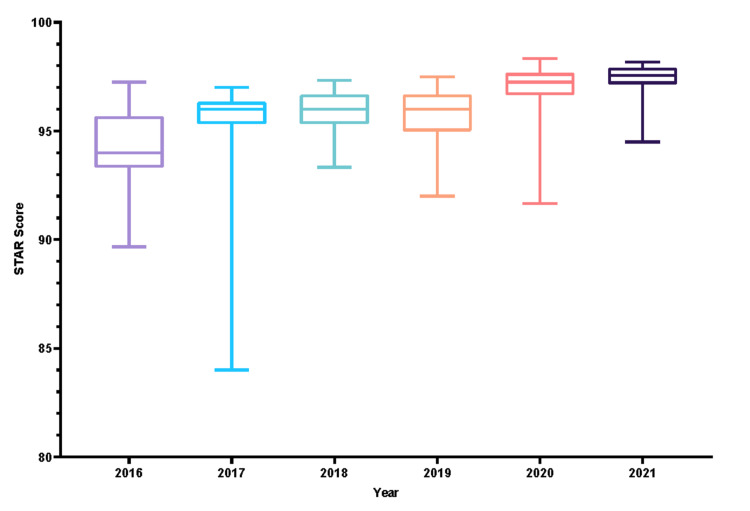
Mean difference across STAR scores STAR: Surgical Tool for Auditing Records

In comparison with the 2016 baseline, the total STAR scores of records of the later years were significantly improved. Nonetheless, the improvement reached a plateau for the years 2017, 2018, and 2019. Present records, as in those documented in 2021, were improved in relation to all the previous years, as their total STAR score was significantly higher than all the other total STAR scores of the earlier years (Figure 1). In regards to the STAR tool’s subsections, significant score changes were initially demonstrated in the Subsequent Entries and Consent domains in 2017. Discharge Summary scores became significantly improved compared to the 2016 baseline starting from 2018. Initial Clerking scores followed a similar pattern among the 2019 records.

**Figure 2:**
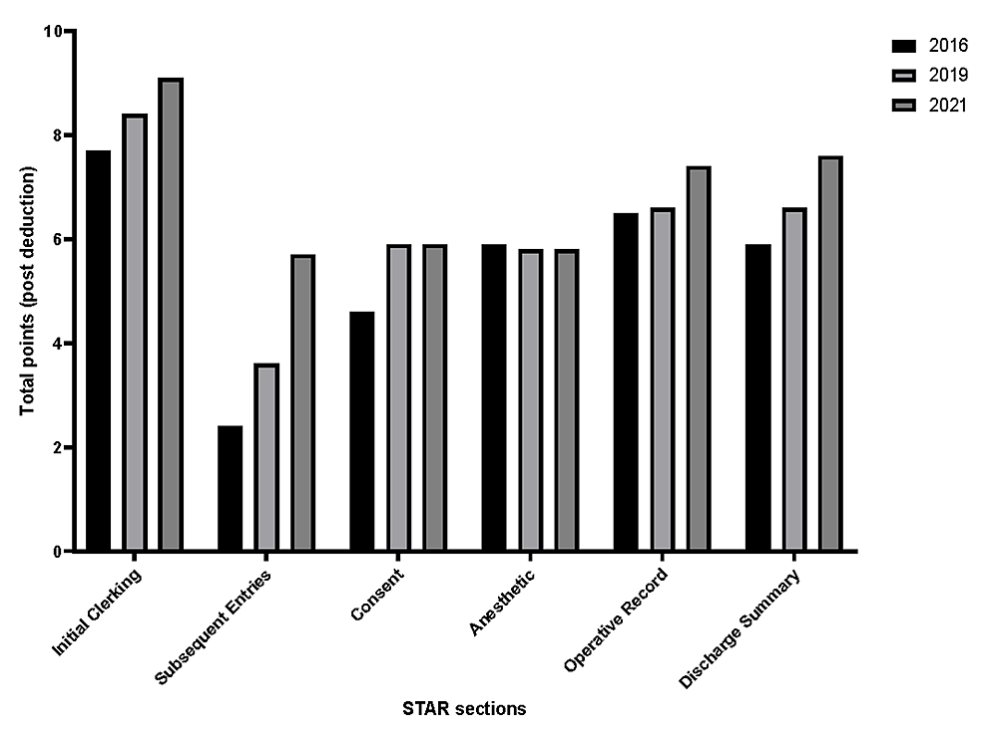
Point deduction across 2016, 2019, 2021

Overall, significant improvements in the STAR scores of the Initial Clerking, Subsequent Entries, and the Operative Record domains were pronounced in the 2021 records. While significantly improved compared to its 2016 baseline, the consent form scores plateaued and did not improve over the years. Additionally, Anesthetic record scores did not demonstrate statistically significant changes all through the study’s timeframe. Figure 2 demonstrates changes in points among the years 2016, 2019, and 2021.

**Figure 3:**
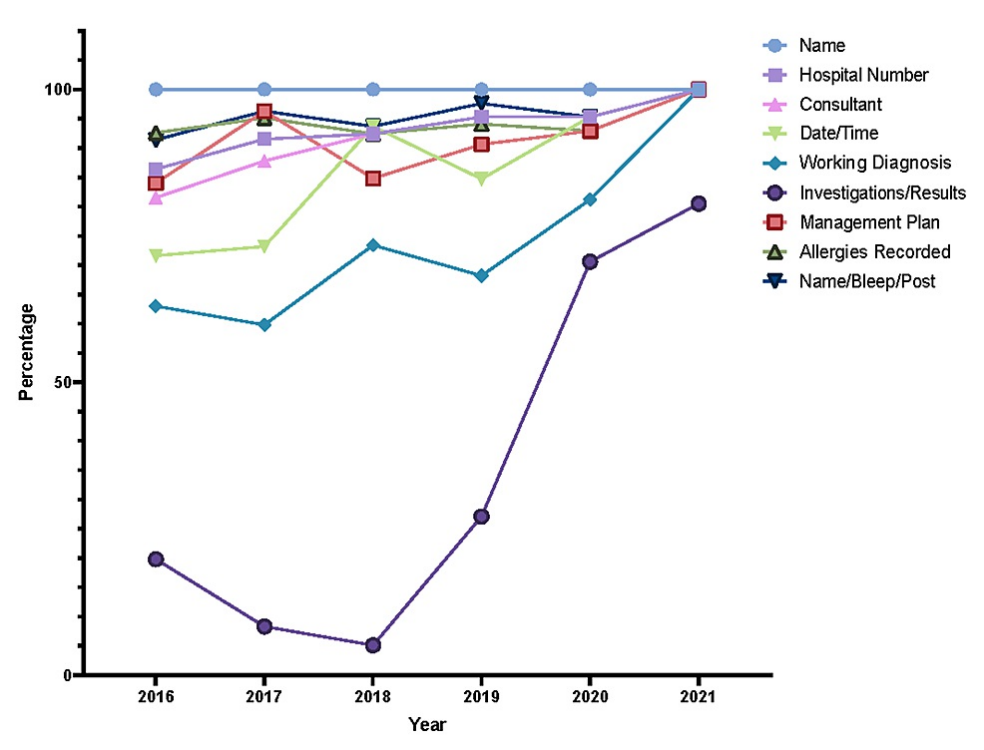
Initial clerking items trends

**Figure 4:**
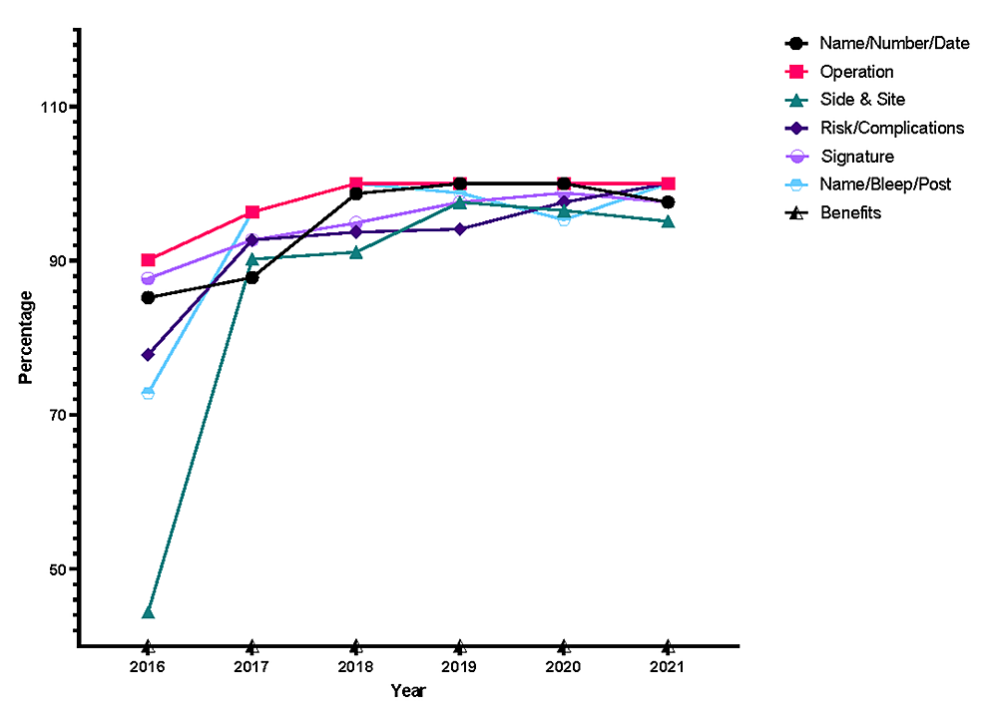
Consent item trends

**Figure 5:**
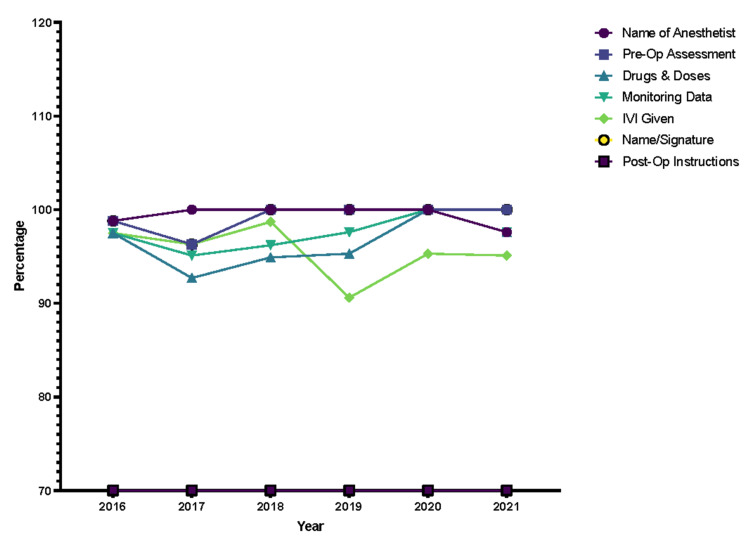
Anesthesia record items trends

**Figure 6:**
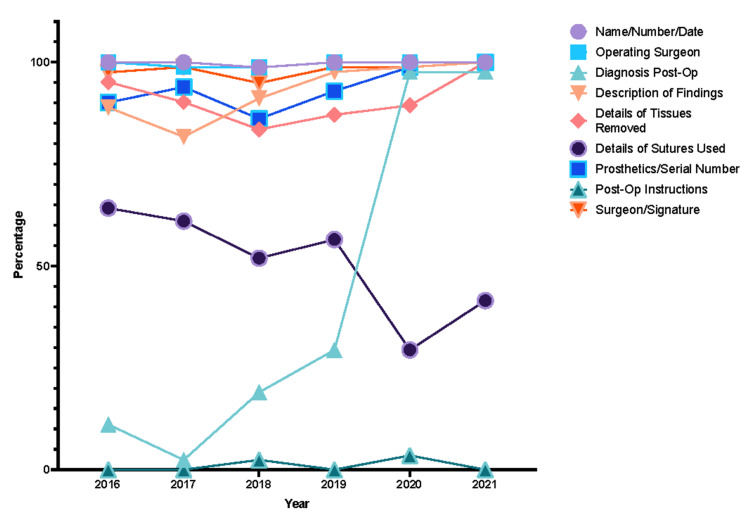
Operative record items trends

**Figure 7:**
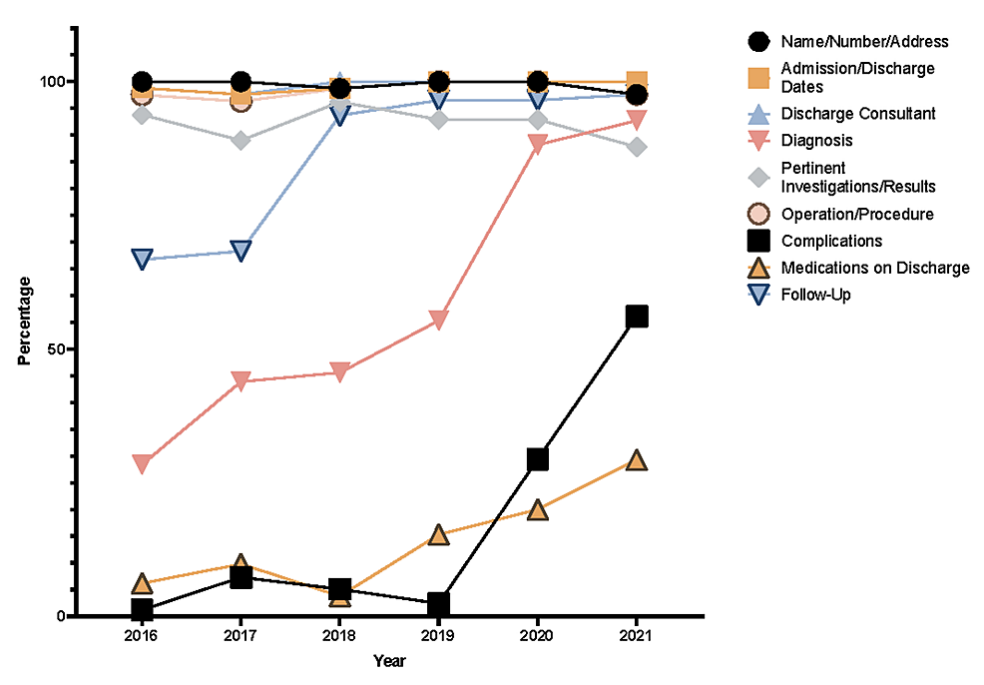
Discharge records items trends

Upon further analysis of each STAR domain, major areas of improvement or lack thereof were noted. Within Initial Clerkship, the investigations/results, working diagnosis, and date/time sub-sections had the greatest margins of improvement (Figure 3). Similarly, the side and site of operation showed a similar trend within the Consent domain (Figure 4). However, points were deducted for benefits, as they were nonexistent in JUH’s consent form throughout the years. The Anesthesia Record domain showed no improvements in all of the subsections (Figure 5) while the Operative Record domain demonstrated major increases in the documentation of postoperative diagnosis (Figure 6). However, within that domain, there were surprising decrements within the reporting of the details of used sutures. Finally, the Discharge Summary domain showed the largest magnitude of record-taking improvement, as four of its subsections (follow-up, diagnosis, complications, and medications on discharge) had significant STAR score increases (Figure 7). It is also noted that postoperative instructions within the Anesthesia Record and Operative Record domains are almost always omitted (Table 2).

**Table 2:** Detailed STAR scores STAR: Surgical Tool for Auditing Records

		2016	2017	2018	2019	2020	2021
Initial Clerking	Name	81 (100%)	82 (100%)	78 (98.7%)	85 (100%)	85 (100%)	41 (100%)
	Hospital number	70 (86.4%)	75 (91.5%)	73 (92.4%)	81 (95.3%)	81 (95.3%)	41 (100%)
	Referral source	66 (81.5%)	72 (87.8%)	73 (92.4%)	81 (95.3%)	81 (95.3%)	41 (100%)
	Consultant	70 (86.4%)	70 (85.4%)	73 (92.4%)	81 (95.3%)	81 (95.3%)	40 (97.6%)
	Date/Time	58 (71.6%)	60 (73.2%)	74 (93.7%)	72 (84.7%)	81 (95.3%)	41 (100%)
	Working diagnosis	51 (63.0%)	49 (59.8%)	58 (73.4%)	58 (68.2%)	69 (81.2%)	41 (100%)
	Investigations/results	16 (19.8%)	6 (7.3%)	4 (5.1%)	23 (27.1%)	60 (70.6%)	33 (80.5%)
	Management plan	68 (84.0%)	79 (96.3%)	67 (84.8%)	77 (90.6%)	79 (92.9%)	41 (100%)
	Allergies recorded	75 (92.6%)	78 (95.1%)	73 (92.4%)	80 (94.1%)	79 (92.9%)	41 (100%)
	Name/Bleep/Post	74 (91.4%)	79 (96.3%)	74 (93.7%)	83 (97.6%)	81 (95.3%)	41 (100%)
Consent Form	Name/Number/Date	69 (85.2%)	72 (87.8%)	78 (98.7%)	85 (100%)	85 (100%)	40 (97.6%)
	Operation	73 (90.1%)	79 (96.3%)	79 (100%)	85 (100%)	85 (100%)	41 (100%)
	Side & site in full words	36 (44.4%)	74 (90.2%)	72 (91.1%)	83 (97.6%)	82 (96.5%)	39 (95.1%)
	Risk/complications	63 (77.8%)	76 (92.7%)	74 (93.7%)	80 (94.1%)	83 (97.6%)	41 (100%)
	Signature	71 (87.7%)	76 (92.7%)	75 (94.9%)	83 (97.6%)	84 (98.8%)	40 (97.6%)
	Name/Bleep/Post	59 (72.8%)	79 (96.3%)	79 (100%)	84 (98.8%)	81 (95.3%)	41 (100%)
	Benefits	0 (0%)	0 (0%)	0 (0%)	0 (0%)	0 (0%)	0 (0%)
Anesthesia Record	Name of anesthetist	80 (98.8%)	82 (100%)	79 (100%)	85 (100%)	85 (100%)	40 (97.6%)
	Pre-op assessment	80 (98.8%)	79 (96.3%)	79 (100%)	85 (100%)	85 (100%)	40 (97.6%)
	Drugs and doses	79 (97.5%)	76 (92.7%)	75 (94.9%)	81 (95.3%)	85 (100%)	40 (97.6%)
	Monitoring data	79 (97.5%)	78 (95.1%)	76 (96.2%)	83 (97.6%)	85 (100%)	40 (97.6%)
	IVI given	79 (97.5%)	79 (96.3%)	78 (98.7%)	77 (90.6%)	81 (95.3%)	39 (95.1%)
	Name/signature	80 (98.8%)	79 (96.3%)	79 (100%)	85 (100%)	85 (100%)	41 (100%)
	Post-op instructions	0 (0%)	0 (0%)	0 (0%)	0 (0%)	0 (0%)	0 (0%)
Operative Record	Name/number/date	81 (100%)	82 (100%)	78 (98.7%)	85 (100%)	85 (100%)	41 (100%)
	Operating surgeon	81 (100%)	81 (98.8%)	78 (98.7%)	85 (100%)	85 (100%)	41 (100%)
	Diagnosis post-op	9 (11.1%)	3 (2.4%)	15 (19.0%)	25 (29.4%)	83 (97.6%)	40 (97.6%)
	Description of findings	72 (88.9%)	67 (81.7%)	72 (91.1%)	83 (97.6%)	84 (98.8%)	41 (100%)
	Details of tissues removed	77 (95.1%)	74 (90.2%)	66 (83.5%)	74 (87.1%)	76 (89.4%)	41 (100%)
	Details of sutures used	52 (64.2%)	50 (61.0%)	41 (51.9%)	48 (56.5%)	25 (29.4%)	17 (41.5%)
	Prosthetics/serial number	73 (90.1%)	77 (93.9%)	68 (86.1%)	79 (92.9%)	84 (98.8%)	41 (100%)
	Post-op instructions	0 (0%)	0 (0%)	2 (2.4%)	0 (0%)	3 (3.5%)	0 (0%)
	Surgeon/signature	79 (97.5%)	81 (98.8%)	75 (94.9%)	84 (98.8%)	84 (98.8%)	41 (100%)
Discharge Summary	Name/number/address	81 (100%)	82 (100%)	78 (98.7%)	85 (100%)	85 (100%)	40 (97.6%)
	Admission/discharge dates	80 (98.8%)	80 (97.6%)	78 (98.7%)	85 (100%)	85 (100%)	41 (100%)
	Discharge consultant	80 (98.8%)	80 (97.6%)	79 (100%)	84 (98.8%)	85 (100%)	41 (100%)
	Diagnosis	23 (28.4%)	36 (43.9%)	36 (45.6%)	47 (55.3%)	75 (88.2%)	38 (92.7%)
	Pertinent investigations/results	76 (93.8%)	73 (89.0%)	76 (96.2%)	79 (92.9%)	79 (92.9%)	36 (87.8%)
	Operation/procedure	79 (97.5%)	79 (96.3%)	78 (98.7%)	85 (100%)	85 (100%)	40 (97.6%)
	Complications	1 (1.2%)	6 (7.3%)	4 (5.1%)	2 (2.4%)	25 (29.4%)	23 (56.1%)
	Medications on discharge	5 (6.2%)	8 (9.8%)	3 (3.8%)	13 (15.3%)	17 (20.0%)	12 (29.3%)
	Follow-up	54 (66.7%)	56 (68.3%)	74 (93.7%)	82 (96.5%)	82 (96.5%)	41 (97.6%)

## Discussion

Our study demonstrated that the quality of surgical notes of patients undergoing elective orthopedic surgeries, as measured by the STAR score, was significantly improved over the past five years. Among all the different domains of the STAR, all but the Subsequent Entries domain exhibited a constant trend toward improving throughout the study’s timeframe. The Consent and Anesthesia Records domains plateaued all through the study’s period. The Consent domain is dictated by a standardized form with minimal input, which might explain the stagnation in its improvement. On the other hand, the Anesthesia Records domain was already of high quality at baseline since it is filled by an entire team dedicated to the anesthetic procedures within the surgical operations.

The quality of surgical notes at JUH is exceptionally high at baseline with significant improvement during the years 2020 and 2021. Our results are similar to that reported by Tuffaha et al., which developed the STAR tool, as a more reliable modification of the CRABEL score, on patients undergoing vascular surgery [21]. In addition, Chalikonda et al. (2018) reported a significant improvement in the quality of surgical orthopedic notes from 76.7% to 81.0% [15]. However, the latter study did not involve any interventions directed toward medical practitioners within their respective institutions but rather demonstrated the effect of adopting an electronic record system on the natural progression and quality of surgical records.

The current study demonstrated that enforcing simple measures, such as educating doctors on appropriate record-keeping practices, periodic and frequent auditing of medical records, and the introduction of dynamic and structured notes are effective in significantly improving the overall quality of medical records. Within JUH, the quality office serves to improve evidence-based practice through annual auditing and the promotion of novel interventions. The effectiveness of such measures was demonstrated throughout literature as the introduction of proformas/aid memoirs, annual auditing, and doctor/student education helped in improving the quality of documentation within surgical records [7,21].

Other interventions, such as the implementation of electronic record systems, which required inputs or the use of dictated notes, can be used as alternatives to ensure higher quality notes [7]. Electronic medical records, while ensuring effective inputting of data, fall prey to a myriad of weaknesses. These weaknesses include the inherent difficulty to create a standalone EMR system, longer time spent for documentation, the introduction of errors, and the need for capital investment and extensive staff training [7,15]. In Jordan, the most dominant EMR system throughout the country’s public, governmental, specialized, and military health institutions is HAKEEM (translates to ‘wise’ in Arabic), which is a modified version of the Veterans Health Information Systems and Technology Architecture (VistA) information system. Nevertheless, the efficiency and effectiveness of the HAKEEM system were never documented nor compared to that of its already established counterparts (e.g., JUH’s EMR) or even traditional paper-based record keeping. This issue applies to most prevalent and global EMR systems, as they are not dynamic, associated with an altered culture of record documentation, or well-integrated enough to provide point-of-care patient services, but rather act as electronic replacements to record archives and cabinets without an active role in patient care [25].

The importance of proper documentation within medical records stems from their use as legal documents [7,15]. Despite this widespread notion, the quality of medical records within medico-legal cases is often variable and insufficient [15]. Furthermore, due to their legal value, especially as more litigation claims are increasing on annual basis among surgical departments [26], administrations of concerned bodies often push for more documentation at the expense of proper healthcare delivery [25]. Moreover, proper documentation is essential as it is a cornerstone in the quality development of hospital services and long-term patient-oriented treatment plans and influences revenue [27]. Nevertheless, the value of documentation throughout healthcare is understudied, as there are no significant efforts delineating the impact of proper documentation on patient care [25].

Throughout the study, almost all evaluated records had deductions due to the absolute lack of a benefits statement within JUH’s standardized consent form and the lack of postoperative instructions in both the operative records and, more importantly, anesthesia records. While the lack of such sections raises considerable concerns, it should be noted that within JUH, its common practice for practitioners to comprehensively discuss surgical operations with their patients. Moreover, the anesthesia team and its associated nursing team are almost exclusively responsible for educating the patients and their legal caretakers about everything they should be aware of in terms of postoperative instructions.

Despite the development of multiple surgical record auditing tools, including the orthopedic-oriented TONK or the STAR score [21,28], these standardized tools were based on a more general CRABEL score and may fail to detect specific concepts that are of major relevance to specific surgical specialties such as the quality of pain assessment [29]. Aside from the scores’ shortcomings and lack of specificity, auditing medical records improves the quality of care, manifested as decreased mortality and more evidenced-based treatments, stimulates continuous development of practitioners through active feedback, improves coding, therefore, stimulating research, and contributes to economic gains, as it promotes more efficient use of resources [29-30]. While the records at JUH are subjected to JCI auditing, the audits are not department-specific nor conducted annually, thus limiting the short-term evaluation of quality improvement interventions.

Our study is subjected to a multitude of limitations. First, the study focused on records within orthopedics, a specialty that is subjected to high rates of litigation and that might have introduced a bias toward better note-taking and documentation. Second, while demonstrating the quality of individual records and notes, the study did not assess the quality of the electronic and paper-based notes themselves. Third, the standardized one-fits-all design of the STAR score may have limited its ability to detect major areas of deficiencies hidden within the vague nature of its concepts, which are often devoid of details. However, our strengths lie in our rigorous methodology, as we had surveyed a random sample of medical records that is average in number, using a well-validated tool within the literature with excellent reliability and low inter-observer variation.

## Conclusions

In summary, our study demonstrated that the quality of surgical notes can be enhanced through simple measures such as teaching medical practitioners and holding periodic auditing. The sustainability and continuous improvement of surgical notes promote high-quality care, polish the practices of medical staff, and increase overall revenue through the promotion of cost-effective healthcare. While auditing tools are appropriate as preliminary evaluation tools for medical records in general, their standardized forms may not be able to detect specialty-specific deficiencies. Therefore, further research should be conducted to develop and validate more comprehensive tools that deviate away from the original CRABEL framework.
